# Is pulmonary Langerhans cell histiocytosis really rare?: A retrospective cohort study

**DOI:** 10.1097/MD.0000000000043766

**Published:** 2025-08-08

**Authors:** Esma Sevil Akkurt, Berna Akinci Ozyurek, Kerem Ensarioglu, Tugce Sahin Ozdemirel, Esma Zenbilli, Hakan Erturk

**Affiliations:** a Department of Chest Disease, Atatürk Sanatorium Research and Training Hospital, University of Health Sciences, Ankara, Turkey; b Department of Radiology, Atatürk Sanatorium Research and Training Hospital, University of Health Sciences, Ankara, Turkey.

**Keywords:** interstitial lung disease, pulmonary Langerhans cell histiocytosis, smoking-related lung diseases

## Abstract

Pulmonary Langerhans cell histiocytosis (PLCH) is a rare interstitial lung disease of unknown etiology, typically affecting individuals aged 20 to 40. Characteristic high-resolution computed tomography findings may obviate the need for biopsy. This study aims to raise awareness by describing clinical and imaging features of PLCH. This single-center retrospective cohort (2016–2024) included 26 confirmed PLCH patients. Demographics, smoking history, comorbidities, symptoms, lab results, 6-minute walk distance, pulmonary function tests, diffusion capacity for carbon monoxide (DLCO), and high-resolution computed tomography findings were reviewed. Median age was 46 years; 84.6% had a smoking history. Cough (46.2%) and dyspnea (53.8%) were common. Radiologically, cysts and nodules predominated. Initial DLCO was lower in dyspneic patients (*P* = .035). Over median 2.5 years of follow-up, pulmonary function tests and DLCO values remained stable. Early recognition of PLCH radiological features and smoking cessation are crucial. Despite limitations, these findings reinforce known patterns and highlight the need for larger, prospective studies.

## 1. Introduction

Pulmonary Langerhans cell histiocytosis (PLCH) is a rare interstitial lung disease of unknown etiology that is most commonly observed in individuals between the ages of 20 and 40 years, characterized by Langerhans cell infiltration in the lungs.^[1]^ However, its true prevalence and incidence are unknown. More than 90% of cases are smokers, suggesting that smoking plays an important role in its pathogenesis.^[2]^

The radiological findings of PLCH contribute significantly to its diagnosis, and these findings can be characteristic. On posteroanterior chest x-rays, there is a bilateral, symmetrical upper, and middle zone-dominant reticulonodular appearance with no costophrenic angle involvement.^[3–5]^ High-resolution computed tomography (HRCT) should be performed in patients who are considered to have PLCH and have these findings on PA chest x-rays. HRCT revealed small nodules with unclear boundaries, cavitary nodules, and thin- and thick-walled cysts. The nodules showed centrilobular distribution. HRCT follow-up studies have shown that nodules transform into cavitary nodules, and then into thick- and thin-walled cysts. Although the definitive diagnosis of PLCH is made by pathological examination, 84% to 90% of correct diagnoses are made by HRCT without biopsy. Therefore, in patients who smoke and have appropriate symptoms, PLCH can be diagnosed based on the presence of typical characteristic radiological findings on HRCT. Lung biopsy is preferred in women with pneumothorax requiring surgical intervention, diffuse cystic lesions, symptomatic nodular lesions for which steroid treatment is prescribed, and in cases with atypical presentation.^[6,7]^ Respiratory function tests remain an acceptable and noninvasive method of patient evaluation, with diagnostic and follow-up value comparison being utilized in most settings.

Owing to the rarity of this disease, the current understanding of it is inadequate, and it is frequently misdiagnosed or missed. This study aimed to increase clinicians’ awareness of this disease and contribute to the literature by outlining the clinical and imaging features of PLCH. Possible correlations between imaging and function tests were also to be investigated regarding their role in PLCH.

## 2. Material and methods

This study included all patients diagnosed with PLCH who were retrospectively screened and consecutively enrolled between January 2016 and March 2024 at our tertiary center specialized in pulmonary medicine. The patients’ clinical information, comorbidities, laboratory values, pulmonary function test (PFT), diffusion capacity for carbon monoxide (DLCO) parameters, 6-minute walking test, radiological imaging results, and prognosis information were recorded retrospectively. Follow-up parameters for PFT and DLCO values were additionally recorded. Missing data from the electronic hospital record system were supplemented using archived physical files. When these sources were also unavailable, the relevant variables were excluded from the analysis. While formal kappa statistics were not computed, HRCT evaluations were independently reviewed by 2 radiologists with over 10 years of experience in thoracic imaging. Discrepancies were resolved through consensus.

This study was approved by the Ethics Committee of the University of Health Sciences Ankara Oncology Training and Research Hospital (Approval No.: 2024-10/149). This research was conducted in accordance with the Declaration of Helsinki (as revised in 2013). Due to retrospective nature of the study, patient consent was not deemed applicable as per ethics’ committee approval.

### 2.1. Statistical evaluation

All parameters included in the study were initially entered into Microsoft Excel Office 365 file for evaluation. After investigating any mistakes, the file was converted, and the following statistical evaluations were performed using an IBM SPSS statistics file (IBM Corp. Released 2017. IBM SPSS Statistics for Windows, Version 25.0. Armonk). The parameters were evaluated for their distribution by histogram, Shapiro–Wilk test, and Q–Q plot analysis, according to the available number of parameters, to confirm the parametric distribution. Afterwards, the values were given by stating the mean and standard deviation if they were parametric, or the median with quartiles if they were nonparametric. Paired sample *t*-tests and Wilcoxon signed-rank test were used to evaluate changes in PFT comparisons, depending on the parametric distribution. Statistical significance was set at *P* < .05. For nonparametric data, the Mann–Whitney *U* and Wilcoxon signed-rank tests were used, while the chi-square or Fisher’s exact test was applied for categorical variables.

## 3. Results

A total of 26 patients were included in this study. The sex distribution was mostly equal, with 14 male participants (53.8%) and 12 female participants (46.2%). The median age was 45.42 (39–56) years. The majority of the patients had a smoking history (n = 22, 84.6%), and chronic obstructive pulmonary disease was the most prevalent comorbidity, observed in 4 (15.4%) patients. Coughing (n = 12, 46.2%) and dyspnea (n = 14, 53.8%) were the most commonly observed symptoms. Sixteen patients (61.5%) were diagnosed radiologically, while the remaining 10 patients had undergone surgery for pathological evaluation; wedge resection (n = 4) and video-assisted thoracoscopic surgery (n = 3) were the most preferred surgical modalities (Table 1).

**Table 1:** Demographic parameters and diagnostic modalities.

Parameters		Count (n = 26, %)
Age (median, 25th to 75th)		45.42 (39–56)
Gender	Male	14 (53.8)
	Female	12 (46.2)
Comorbidity	Diabetes mellitus	2 (7.7)
	Asthma	2 (7.7)
	COPD	4 (15.4)
	Rheumatoid arthritis	1 (3.8)
	Sarcoidosis	1 (3.8)
Dyspnoea	None	12 (46.2)
	Present	14 (53.8)
Coughing	None	14 (53.8)
	Present	12 (46.2)
Pleuritic pain	None	23 (88.5)
	Present	3 (11.5)
Smoking history	None	4 (15.4)
	Present	22 (84.6)
Smoking package (median, 25th to 75th)		25.22 (17–30)
Diagnostic method	Pathological	10 (38.5)
	Radiological	16 (61.5)
Surgery type	Wedge resection	4 (44.4)
	VATS	3 (33.3)
	Thoracotomy	1 (11.1)
	EBUS	1 (11.1)

COPD = chronic obstructive pulmonary disease, EBUS = endobronchoscopic ultrasonography, VATS = video-assisted thoracoscopic surgery.

Cysts (n = 24, 92.3%) and nodules (n = 20, 76.9%) were the most common findings in radiological evaluations of the patients, with fibrosis (n = 4, 15.4%), cavities (n = 4, 15.4%), and ground glass (n = 3, 11.5%) being reported relatively rarely. Pneumothorax was observed in 2 patients. Regarding lung zone involvement, the costophrenic angle and upper zones were spared in all the patients. Lower zone involvement was also less frequent (n = 12, 46.2%) than in the middle zone, and all but 1 patient was affected (96.2%; Table 2).

**Table 2:** Radiological findings and lung zone involvement distribution of patients.

Parameters		Count (n = 26, %)
Cysts	None	2 (7.7)
	Present	24 (92.3)
Nodule	None	6 (23.1)
	Present	20 (76.9)
Fibrosis	None	22 (84.6)
	Present	4 (15.4)
Cavity	None	22 (84.6)
	Present	4 (15.4)
Ground glass	None	23 (88.5)
	Present	3 (11.5)
Pneumothorax	None	24 (92.3)
	Present	2 (7.7)
Lung zone involvement comparison		
Costophrenic angle	None	26 (100)
	Present	0 (0)
Upper zone	None	0 (0)
	Present	26 (100)
Middle zone	None	1 (3.8)
	Present	25 (96.2)
Lower zone	None	14 (53.8)
	Present	12 (46.2)

The patients were evaluated for symptoms, radiological findings, respiratory function, and laboratory test results. Coughing, dyspnea, and lower zone involvement were included in the analysis. In the nonparametric comparison between groups, the only difference was observed in the patients with dyspnea regarding the initial DLCO value, with dyspnea observed more in patients with a lower DLCO (*P* = .035). However, owing to the limited number of patients, the statistical reliability was deemed low (Table 3).

**Table 3:** Nonparametric comparison between groups.

Parameters (n, mean rank)	Coughing			Dyspnoea			Lower zone involvement		
	Present	None	*P* value	Present	None	*P* value	Present	None	*P* value
Initial FVC	10 (8.9)	7 (9.14)	1	9 (9.56)	8 (8.38)	.673	9 (9.44)	8 (8.5)	.743
Follow FVC	6 (5.58)	6 (7.42)	.394	6 (6.75)	6 (6.25)	.818	6 (4.92)	6 (8.08)	.132
Initial DLCO	7 (5.29)	6 (9)	.101	7 (9.14)	6 (4.5)	.035	7 (8.93)	6 (4.75)	.051
Follow DLCO	5 (3.40)	4 (7)	.063	5 (5.4)	4 (4.5)	.73	4 (6)	5 (4.2)	.413
Initial DLCO/VA	6 (5.33)	6 (7.67)	.31	7 (7.43)	5 (5.2)	.343	7 (7.93)	5 (4.50)	.106
Follow DLCO/VA	5 (3.6)	6 (7.5)	.111	5 (5.80)	4 (4)	.413	4 (6.5)	5 (3.8)	.19
RDW	14 (14.29)	12 (12.58)	.595	12 (11.21)	14 (15.46)	.16	14 (14.32)	12 (12.54)	.56
Platelets	14 (11.79)	12 (15.50)	.231	12 (15.25)	14 (12)	.297	14 (13.0)	12 (14.08)	.742
Neutrophil/lymphocyte	14 (14.86)	12 (11.92)	.347	12 (13.33)	14 (13.64)	.94	14 (12.86)	12 (14.25)	.667
Lymphocyte/platelet	14 (13.36)	12 (13.67)	.94	12 (10.54)	14 (16.04)	.067	14 (14.36)	12 (12.5)	.56

DLCO = diffusing capacity of the lung for carbon monoxide, FVC = forced vital capacity, RDW = red cell distribution width, VA = alveolar volume.

## 4. Discussion

PLCH is a rare interstitial lung disease with an unknown cause that is most commonly seen in people between the ages of 20 and 40 years, in which Langerhans cell infiltration is observed in the lungs.^[8]^ PLCH should be considered in young smokers who present with spontaneous pneumothorax and more widespread reticular, reticulonodular, and cavitated nodular infiltration in the upper and middle areas radiologically. The elimination of cigarette smoking is the cornerstone of the management of patients with PLCH. Since studies on this rare disease in our country are limited, we believe that this study will make an important contribution to the literature despite the small sample size.

PLCH is an inflammatory myeloid neoplasm characterized by abnormal accumulation of dendritic cells harboring activating mutations in the mitogen-activated protein kinase pathway.^[8]^ There is a strong association between PLCH and cigarette smoke exposure, and more than 90% of patients with PLCH are current or former smokers.^[9]^ A recent mouse model of PLCH provided evidence that cigarette smoke exposure may increase the accumulation of myeloid cells harboring mitogen-activated protein kinase mutations in the lungs.^[10]^ In our study, the majority of patients had a history of smoking (n = 22, 84.6%), and the most common concomitant disease was chronic obstructive pulmonary disease, which occurred in 4 (15.4%) patients. The most common symptoms reported in literature are nonproductive cough, shortness of breath, and chest pain. There were no symptoms in 25% of cases, and the diagnosis was made by chance or after a noisy picture, such as spontaneous pneumothorax, developed by examining the pathological findings on chest x-rays obtained for other reasons.^[11]^ In our study, cough and dyspnea were the most common symptoms, and pneumothorax was observed in 2 patients.

Characteristic chest CT findings of PLCH include nodules, thin-walled cysts, and/or thick-walled cavities. Disease characteristics depend on the stage at which the disease is detected. In the initial stages, chest imaging primarily consists of numerous nodules that develop into thick-walled cavities and thin-walled cysts over time. The cysts in PLCH are generally irregularly shaped and appear as tubular or branching structures. Radiological findings in PLCH are more pronounced in the middle to upper lung regions, where the costophrenic sulci are spared.^[12]^ In our study, the most frequently reported findings in the radiological evaluation of patients were cysts (92.3%) and nodules (76.9%), followed by fibrosis (15.4%), cavities (15.4%), and ground glass (11.5%) were reported relatively rarely. In terms of lung region involvement, the costophrenic angle and upper regions were preserved in all the patients. Lower region involvement was also observed less frequently than in the middle region (46.2%), and all patients (except 1) were affected compared with the middle region (96.2%).

The prevalence and incidence of PLCH are difficult to estimate. A large series of surgical lung biopsies in patients with interstitial lung disease detected PLCH in 5% of samples.^[13]^ However, many patients with PLCH do not undergo surgical lung biopsy for various reasons. In some patients, the disease is never suspected, while others are diagnosed based on radiological features observed on HRCT of the lungs. In 1 study, PLCH was diagnosed in 91 (6.6%) of the 1382 patients included in the Italian interstitial lung disease registry.^[14]^ This study supports the evaluation of patients diagnosed by clinical and radiological studies without biopsy confirmation. Sixteen patients (61.5%) were diagnosed radiologically, whereas the remaining 10 patients underwent surgery for pathological evaluation, of which wedge resection (n = 4) and video-assisted thoracoscopic surgery (n = 3) were the most preferred surgical methods.

Delobbe et al performed a survival analysis of 45 patients with PLCH to determine survival predictors. The patients were 28 ± 10 years of age, divided into 32 male and 13 female participants, almost smoked (96%), and had symptoms at the time of diagnosis (78%). Diagnoses were made using lung biopsy in 25 patients (56%) and bronchoalveolar lavage analysis in 20 patients (44%). Patients were followed-up for a median of 6 years after diagnosis, and 33 (73%) patients survived during the observation period. The present findings suggest that older age, lower forced expiratory volume (FEV1)/forced vital capacity (FVC) ratio, and higher residual volume (RV)/total lung capacity (TLC) ratio are adverse prognostic factors at the time of diagnosis of PLCH.^[15]^ Another study, conducted in 2015, evaluated 40 patients with PLCH. The mean age of the patients was 40 ± 14 years, and 22 of them were women. The diagnosis was based on the search for CD1a+cells in bronchoalveolar lavage (10 patients), lung biopsy (8 patients), or biopsy of cystic bone lesions (2 patients). In 12 patients, the diagnosis was based on clinico-radiological data.

Regarding treatment management, smoking cessation was the mainstay treatment for 25 patients. Prednisolone and vinblastine treatments were reserved for patients with major pulmonary or extrapulmonary involvement.^[16]^ PLCH, as a subtype of LCH, has been reported to be consisted of 2 distinct groups, with 1 favoring extrapulmonary involvement.^[17]^

Multisystemic involvement in LCH has been known to be treated with combination of cytotoxic (vinblastine or in rare cases, methotreaxate) and glucocorticoid therapy, however evidence remains limited regarding isolated PLCH. Glucocortiod therapy has been used on empirical grounds, and with regimens of 0.5 to 1 mg/kg/d, tapered over 6 to 12 months, however evidence-based data also remains limited in this usage.^[13]^ In a study in China that retrospectively analyzed the clinical and follow-up data of 15 PLCH cases, there were 3 patients with single-system PLCH and 12 patients with multisystem PLCH. The most common CT findings in this patient cohort were nodules and/or cysts, and 7 patients had nodular and cystic shadows. Three patients had simple multiple cystic shadows, 3 patients had multiple nodules and 2 patients had single nodules and mass shadows. Patients with PLCH with a history of smoking were advised to quit smoking. Ten patients received chemotherapy, while 2 received oral glucocorticoid therapy.^[18]^ In our study, only 1 patient had required a short term treatment with corticosteroid due to respiratory distress, however, the total treatment duration was within a month. Other patients did not have any immunosuppressive treatment regimens. As such, while mentioned treatment modalities are available, we could not report our experience regarding outcome of these management strategies.

The PFTs are highly variable during PLCH. Evidence of airway obstruction is common and usually characteristic of the disease. FEV1, FVC, RV, TLC, DLCO, and alveolar volume are the main parameters utilized in PFT for disease evaluation. Decreased FEV1/FVC ratio and increased RV/TLC ratio are common findings, especially in patients with extensive cystic lesions. In the early stages, granulomas tend to form around the bronchioles and are associated with mild restrictive changes and/or a decrease in DLCO. In the end stage, cystic lesions of variable sizes are present, and PFT often show evidence of airflow obstruction and air retention. In our study, the median follow-up period was 928.5 (304–2373 days). The median FEV1, FVC, FEV1/FVC ratio, DLCO, and DLCO/alveolar volume were 89%, 94%, 75.5%, 71%, and 84%, respectively, and there were no statistically significant differences according to follow-up values.

Symptoms and radiological findings were evaluated in terms of their roles in respiratory function and laboratory test results. Cough, dyspnea, and lower region involvement were included in the analysis. In the nonparametric comparison between the groups, the only difference observed was in patients with dyspnea in terms of the initial DLCO value. Dyspnea was observed more frequently in the patients with lower DLCO values. However, the statistical reliability was considered low because of the limited number of patients included in this study.

Limitations of the study mainly arised from the patient count. As stated earlier, while correlations were indeed present between symptoms and PFT results, a statistical reliability could not be stated due to nonparametric evaluation. Rarity of the disease, combined with imaging modalities being utilized when required, also made radiological comparison over a longitudinal period difficult. Further studies, while still would be limited by the nature of the disease, may provide different clinical information regarding PLCH, especially for those undergoing additional immunosuppressive treatment.

## 5. Conclusion

We believe that this is a valuable study because PLCH is a rare and frequently overlooked condition. Owing to the high spontaneous remission rate, there are no reliable data on the effectiveness of various treatment programs on survival. If an early diagnosis is made and patients are encouraged to quit smoking as soon as possible, a good prognosis will be observed; therefore, awareness by physicians is important. Future studies and prospective multicenter trials should evaluate whether the identification of clinical, functional, and radiological factors, including HRCT findings at the time of diagnosis and during the first year of follow-up, can provide useful information for the management and treatment of PLCH.

## Acknowledgments

The authors thank all the patients who participated in this study. This study was presented in abstract form at the TUSAD Solunum 2024 Congress (Abstract ID: SS-013). The manuscript contains original, unpublished material and is not a copyright violation.

## Author contributions

**Conceptualization:** Esma Sevil Akkurt, Berna Akinci Ozyurek.

**Data curation:** Esma Sevil Akkurt, Kerem Ensarioglu, Esma Zenbilli.

**Formal analysis:** Kerem Ensarioglu, Esma Zenbilli, Hakan Erturk.

**Methodology:** Esma Sevil Akkurt, Berna Akinci Ozyurek, Tugce Sahin Ozdemirel, Hakan Erturk.

**Project administration:** Esma Sevil Akkurt, Berna Akinci Ozyurek, Tugce Sahin Ozdemirel.

**Resources:** Kerem Ensarioglu.

**Software:** Kerem Ensarioglu, Hakan Erturk.

**Supervision:** Esma Sevil Akkurt, Berna Akinci Ozyurek.

**Validation:** Tugce Sahin Ozdemirel.

**Visualization:** Esma Sevil Akkurt, Kerem Ensarioglu, Hakan Erturk.

**Writing – original draft:** Esma Sevil Akkurt.

**Writing – review & editing:** Berna Akinci Ozyurek, Tugce Sahin Ozdemirel.
